# Utilization of the Behavior Change Wheel framework to develop a model to improve cardiometabolic screening for people with severe mental illness

**DOI:** 10.1186/s13012-017-0663-z

**Published:** 2017-11-14

**Authors:** Christina Mangurian, Grace C. Niu, Dean Schillinger, John W. Newcomer, James Dilley, Margaret A. Handley

**Affiliations:** 1 Department of Psychiatry, Weill Institute for Neurosciences, UCSF at Zuckerberg San Francisco General (ZSFG), 1001 Potrero Avenue, 7M8, San Francisco, CA 94110 USA; 2 UCSF Center for Vulnerable Populations at ZSFG, San Francisco, CA USA; 3 UCSF Department of Medicine, Division of General Internal Medicine at ZSFG, 1001 Potrero Avenue, 1320A, San Francisco, CA 94110 USA; 4 Department of Clinical Biomedical Sciences, Charles E. Schmidt College of Medicine, Florida Atlantic University, 777 Glades Road, BC-71 Rm 241, Boca Raton, FL 33431 USA; 5 UCSF Department of Epidemiology and Biostatistics, 550 16th Street, San Francisco, CA 64158 USA

**Keywords:** Cardiometabolic screening, Severe mental illness, Behavior change wheel

## Abstract

**Background:**

Individuals with severe mental illness (e.g., schizophrenia, bipolar disorder) die 10–25 years earlier than the general population, primarily from premature cardiovascular disease (CVD). Contributing factors are complex, but include systemic-related factors of poorly integrated primary care and mental health services. Although evidence-based models exist for integrating mental health care into primary care settings, the evidence base for integrating medical care into specialty mental health settings is limited. Such models are referred to as “reverse” integration. In this paper, we describe the application of an implementation science framework in designing a model to improve CVD outcomes for individuals with severe mental illness (SMI) who receive services in a community mental health setting.

**Methods:**

Using principles from the theory of planned behavior, focus groups were conducted to understand stakeholder perspectives of barriers to CVD risk factor screening and treatment identify potential target behaviors. We then applied results to the overarching Behavior Change Wheel framework, a systematic and theory-driven approach that incorporates the COM-B model (capability, opportunity, motivation, and behavior), to build an intervention to improve CVD risk factor screening and treatment for people with SMI.

**Results:**

Following a stepped approach from the Behavior Change Wheel framework, a model to deliver primary preventive care for people that use community mental health settings as their de facto health home was developed. The CRANIUM (cardiometabolic risk assessment and treatment through a novel integration model for underserved populations with mental illness) model focuses on engaging community psychiatrists to expand their scope of practice to become responsible for CVD risk, with significant clinical decision support.

**Conclusion:**

The CRANIUM model was designed by integrating behavioral change theory and implementation theory. CRANIUM is feasible to implement, is highly acceptable to, and targets provider behavior change, and is replicable and efficient for helping to integrate primary preventive care services in community mental health settings. CRANIUM can be scaled up to increase CVD preventive care delivery and ultimately improve health outcomes among people with SMI served within a public mental health care system.

## Background

People with severe mental illness (SMI—e.g., schizophrenia, bipolar disorder) die on average 25 years earlier than the general population, most often from cardiovascular disease (CVD) [1, 2]. Although CVD mortality in this population is multifactorial, some risk is attributed to treatment with antipsychotic medications [3]. In 2004, the American Diabetes Association and American Psychiatric Association published metabolic screening guidelines for people taking antipsychotic medications [4]. Although adherence to screening guidelines improves care in the general population [5], a review of 48 studies on metabolic monitoring of people taking antipsychotic medications found screening to be consistently low [6]. Leading experts agree that the geographic, electronic, cultural, and fiscal separation of primary care and mental health within most US healthcare systems contributes to poor quality of medical care in general for this vulnerable population [3, 7]. Notably, people with SMI *are* receptive to treatment of metabolic disorders when available as there is evidence that adherence to hypoglycemic medications is better among diabetes patients with schizophrenia than those without [8–10]. Given the complexity of this problem, public mental health administrators request cost-effective, evidence-based interventions that can be feasibly implemented and sustained to improve care for this vulnerable population [3, 11].

Fortunately, integration of behavioral and physical health care has become a significant focus of recent reforms [12]. This trend is critical given that behavioral health conditions account for nearly 25% of all disabilities worldwide [13], mental and physical health disorders are strongly associated [14], and patients experiencing comorbid behavioral health conditions and medical disabilities are costly to the health care system [15], and most people with mental illness lack formal treatment [16]. Collaborative Care is an evidence-based model for integrating mental health, behavioral health, and substance use services with primary care settings [17]. This team-based model has four main principles: (1) patient-centered team care (a primary care provider, behavioral health care manager, and consulting psychiatrist), (2) population-based care (patient registry), (3) measurement-based treatment to target (serial PHQ-9 screening), and (4) evidence-based care (guideline-recommended psychotherapies and pharmacological management of depression). In a recent Cochrane review, Collaborative Care was reported to demonstrate efficacy and cost-effectiveness in over 80 randomized controlled trials [18].

Unfortunately, structural dysfunctions in the US public healthcare system—where mental health and primary care exist in separate silos—are major contributors to disparities along the health care continuum [19, 20]. Although CVD risk factor screening could occur in primary care, people with SMI have significantly lower utilization of primary care than the general population [21–24]. However, almost half of the people with SMI regularly access community mental health services, making these settings the de-facto “health home” for 3.5 million people with SMI [25–28]. A health home is a team-based clinical approach that aims to improve outcomes and experience of care, as well as to control costs, through coordinated care and linkages to community supports for individuals with multiple chronic conditions [29]. “Reverse integration”—provision of primary care in community mental health clinics— initiatives are reasonable considering the parallel in primary care [18]. Four arguments for screening and treating depression in primary care are as follows: (1) major depression is common in general medical settings [30], (2) people who are depressed are likely to be receiving care from a primary care provider [31], (3) depression treatment guidelines exist [32], and (4) untreated depression is costly to society [33]. Parallel arguments justify screening and treatment in community mental health: (1) CVD risk factors (e.g., diabetes, hypertension, dyslipidemia, smoking) are common among people with SMI [20, 34, 35], (2) people with SMI receive care in community mental health settings [25, 26], (3) treatment guidelines for CVD risk factors exist [36–39], and (4) untreated CVD risk factors are costly to society [40].

While several reverse integration models have been proposed [23, 25, 41–45], Cochrane, RAND, and other systematic reviews have failed to identify sufficient evidence to yet recommend any specific model [46–48]. Several excellent NIH-funded investigators and SAMHSA-funded administrators have been working hard to develop models to improve the health care of people with SMI. For example, investigators have conducted trials studying satellite primary care clinics [23], peer-led medical disease self-management [49–51], electronic reminders to providers or patients [52], a clinical decision support program for primary care providers treating people with SMI [53, 54], care coordination [23, 41–43, 45], provider education [55], and other patient-centered approaches [44, 47, 56–58]. *None of these studies target provider behavior by expanding the scope of practice of community psychiatrists.*

While most experts agree in theory that a “behavioral health home”—an integrated care model for people with severe mental illness, based on the conceptual model of a health home and located in community mental health settings—would be the best approach for this population [59–61], to our knowledge, no one has explicitly utilized implementation science methods to develop an effective and sustainable model of care where psychiatrists take primary responsibility for the medical care of their patients.

This paper describes the use of an implementation science framework—the Behavior Change Wheel [62]—to develop an integration of care model for people with SMI served in community mental health settings. We chose this model because our main target was changing psychiatrists’ behavior so they would address primary preventative services for people with SMI. Although the BCW framework is not new, we believe that readers might benefit from an applied and innovative example of in this unique setting. To our knowledge, this study is innovative because the vast majority of integration of care community programs have not been using behavioral change theory approaches in the development of their models [61].

## Methods

### Theoretical frameworks used to develop the model

To develop a model to improve the CVD risk factor screening and treatment of people with SMI served in community mental health clinics, we decided to systematically approach the problem using a specific implementation science framework to guide the process of the intervention development. We wanted to use this systematic approach so that the intervention we developed would be well grounded in behavior change theory such that specific behavioral targets could be first identified and then addressed with intervention components mapping to the targets. We drew primarily on Michie and colleagues’ Behavior Change Wheel (BCW) framework [62] and the associated and the Theoretical Domains Framework (TDF) [63, 64], as well as on, the Theory of Planned Behavior (TPB) [65]. The BCW framework and TDF were chosen as they link identified behavioral targets with to intervention functions most likely to bring about clinic- and provider-level change. The TPB has been widely used in settings focusing on provider behaviors and was selected to complement the BCW and TDF approaches for improving the understanding of behavior change “targets” required to ensure that people with SMI receive metabolic screenings and treatment at a community mental health clinic. This integrated approach to exploring the barriers to delivery of a cardiometabolic screening model to patients with SMI and subsequent intervention development through multiple theoretical lenses allows for us to understand in more depth how intervention components ‘map back’ to several important underlying theoretical constructs. For example, this approach allows for a rich examination of the cognitively oriented role of provider attitudes, as in the TPB, while at the same time also being able to examine a wider range of factors underlying the BCW approach, which includes a greater focus on non-cognitive factors.

**Fig. 1:**
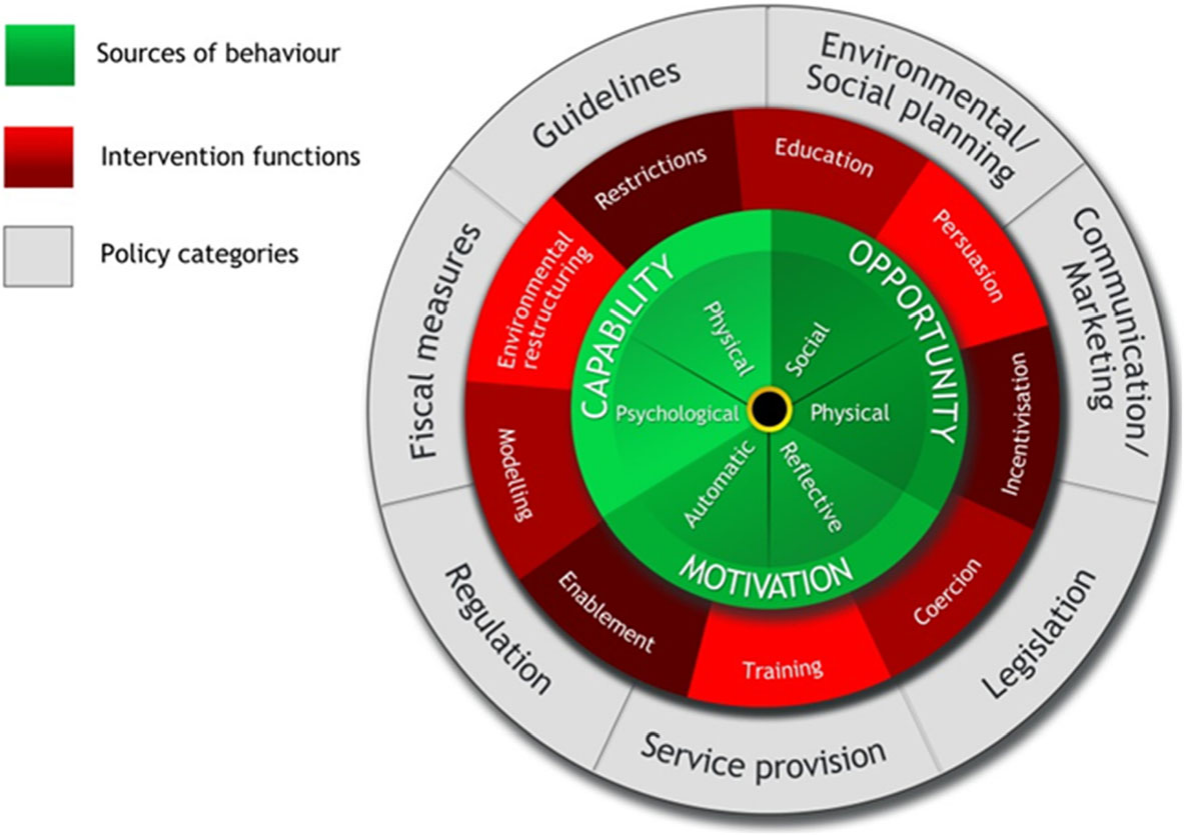
The Behavior Change Wheel [62]

The BCW framework consists of a behavior system at the hub with three critical components: capability, opportunity, and motivation (COM-B model) (see Fig. 1). Surrounding the hub are nine intervention functions that aim to address the deficits in one or more of these conditions. A larger wheel surrounds the intervention functions and consists of seven policy categories. These policy categories are broader population-level strategies that enable the intervention functions to occur.

The Theory of Planned Behavior (TPB) has been widely used to understand social and health behaviors and to develop interventions [65]. The TPB proposes that an individual’s intention to perform a behavior is the best predictor of behavior and suggests that there are three immediate determinants of intention. These include an individual’s attitude regarding the targeted behavior, their subjective norm regarding the behavior, and their perceived behavioral control over the behavior [65]. The TPB relates directly to the COM-B model (capability, opportunity, and motivation are the key ingredients of behavior change) in that motivation is analogous to intention and is also further influenced by social norms while capability is influenced by perceived behavioral control. Furthermore, studies suggest that this theory has demonstrated success in improving various health behaviors [66].

The TDF is related to the BCW and was created from numerous behavior change theories, including the TPB, through a consensus process [62–65]. The framework includes 14 domains: knowledge; skills; memory; attention and decision processes; behavioral regulation; social role and identity; beliefs about capabilities; optimism; beliefs about consequences; intentions; goals; reinforcement; emotion; environmental context and resources; and social influences [47]. Each domain is related to a set of theoretical constructs that was derived from existing theories of behavior change [35]. Since each domain of the TDF correlates to a BCW COM-B component, using the two together allows for an expansion of the COM-B components into very specific domains [67]. We chose BCW and TDF instead of other implementation models (e.g., CFIR) because of their strong emphasis on behavior change, and the utilization of these framework in development of several other interventions targeting health care professionals’ behavior [68–76]. There are few examples of using the selected implementation science frameworks for integration of multiple components of care in a context of co-management across disciplines. For example, in a recent systematic review of changing healthcare professionals’ behavior [68], only one study focused on integration of care [77], but this study focused on case managers—not physicians—and lacked formal identification of barriers and linking of barriers to components. In a recent review of using theory to plan or evaluate guideline implementation among physicians [76], we found no other studies focusing on guideline adherence that requires integration of care and co-management. This paper adds value to understand the utility of using such frameworks when planning integration of care activities across medical disciplines.

### Procedures

We followed the eight steps outlined by the BCW framework, specifically: (1) define the problem to be addressed in behavioral terms; (2) select the target behavior(s) most likely to bring about change to address the problem; (3) specify the target behavior in as much detail as possible [78]; (4) identify what needs to shift in order to achieve the target behavior; (5) identify intervention functions; (6) identify policy categories; (7) identify behavioral change techniques; and (8) identify mode of delivery. The authors reviewed the detailed guidance available on how to approach the tasks needed for each step according to the BCW with reference to the underlying evidence [62].

Since a key aspect of the BCW is gleaning information from stakeholders, we began our model development process by conducting a series of focus groups to inform each step of the model. The stakeholder focus groups consisted of 6–8 participants who were either consumers with severe mental illness, providers, and healthcare administrators. For the consumer focus group, we sought to recruit a racial/ethnically diverse sample reflected the diversity in patients who receive services from one San Francisco Health Network (SFHN) community mental health clinic. The two provider focus groups consisted of community psychiatrists and six primary care physicians working in either a SFHN or an SFHN-affiliated clinic and spent at least 5% of their time treating adults with SMI. For the administrator focus group, we invited all directors of primary care outpatient services, behavioral health outpatient services, pharmacy, and information technology from SFHN to participate and provide input on metabolic screenings and treatment. All subjects provided written informed consent to participate in the focus group as approved by the UCSF IRB (12-09789), and all focus groups were audio-recorded and transcribed for data analysis [79]. To gather data on beliefs and attitudes around the barriers to metabolic screening and acceptability of the expansion of psychiatrists’ scope of practice, the content of the focus group centered on (1) metabolic screening; (2) treatment of metabolic abnormalities; and (3) acceptability of potentially expanding the scope of practice of psychiatrists in community mental health clinics (see Appendix A for full interview guide).

Data from the focus groups were initially used to identify relevant TPB domains and constructs in three areas (attitudes, subjective norms, and perceived behavioral control) that would influence provider behavior. Then, for each step of the BCW framework, two members of the research team were jointly responsible for analyzing, summarizing, and combining focus group findings, peer-reviewed literature, and their own clinical experience. Any disagreements were discussed until consensus was reached. Final decisions were reviewed by the larger research team and confirmed by a research community advisory group that included a medical director and several psychiatrists and social workers at one large community mental health clinic.

## Results

### Participants

Four focus groups were conducted with each of the following stakeholder groups: consumers with severe mental illness (*n* = 8), psychiatrists (*n* = 8), primary care providers (*n* = 6), and healthcare administrators (*n* = 7). Appendix B includes participant demographic information.

### Step 1: Define the problem to be addressed in behavioral terms

Although multiple factors within the healthcare delivery system contribute to the poor medical care of people with SMI, the first step according to the BCW model is to identify a specific problem and define it in behavioral terms. As outlined in the introduction, most of the early morbidity and mortality among people with SMI is from cardiovascular disease. Our research team has been focused on improving detection of cardiometabolic risk and knows the evidence that less than 30% of patients with SMI receive guideline-recommended metabolic screenings [80]. Given our prior research, the specific problem we identified was low metabolic screening in community mental health clinics. We then defined the behavioral target to be the ordering of annual metabolic screening labs (e.g., hemoglobin A1c and lipid panel). The BCW requires identification of the performers of the task and the place in which they will be performing the task. Our focus group findings indicated that community psychiatrists felt responsible for metabolic screenings [81]. Furthermore, people with SMI use community mental health services more often than primary care services [82], often visit their psychiatrist several times a year, and some consider community mental health to be this populations’ medical home [83]. Thus, we specified psychiatrists as the “performers,” community mental health clinics as the “place,” and ordering metabolic screening labs for people with SMI as the “behavioral target.”

### Step 2: Select the target behaviors most likely to bring about change to address the problem

Since behaviors do not occur in isolation but rather in a system, a key task was to understand the context of other behaviors relevant to the target behavior of psychiatrists working at community mental health clinics ordering and managing metabolic screenings. To do this, using data from the focus groups and our review of the literature, our research team generated a list of potential target behaviors that addressed the problem of low metabolic screening in community mental health clinics. This list included engaging patients to be more responsible for their metabolic screening labs, engaging family members to request screenings, having primary care providers take enhanced responsibility for understanding the metabolic risks, developing a system-wide metabolic screening effort, and having psychiatrists assume the role of ordering cardiometabolic labs. Next, we organized and prioritized all the potential target behaviors to determine which behavior was dependent on other behaviors, which behaviors were relevant to be performed by the target group and ultimately which behavior or set of behaviors to intervene. We then used a list of BCW criteria to estimate the likelihood and promise of each potential targeted behavior. These criteria involved assessing the likely impact of the behavior change, the likelihood of the change actually occurring based on the capability, opportunity and motivation of the target group, the spillover effect of the behavior change, and the ease of measuring the behavior. Two members of the research team independently reviewed the various criteria, and any disagreements were resolved through discussion and group consensus. The prioritization process maintained the target behavior identified in Step 1: Community psychiatrists ordering annual metabolic labs for their patients with SMI served in community mental health clinics.

### Step 3: Specify the target behavior in as much detail as possible

In this step, we used specific BCW questions to guide the development of the process for carrying out the target behavior:

- Who needs to perform the behavior? Psychiatrists or nurse practitioners.
- What do they need to do differently to achieve the desired change? Regularly assess whether patients are due for annual metabolic screening rather than defer this to primary care.
- When do they need to do it? When seeing patients taking antipsychotic medications
- Where do they need to do it? At the community mental health clinic.
- How often do they need to do it? Annually for each patient (likely about 10–15 times a month given a typical caseload for a full-time community psychiatrist.
- With whom do they need to do it? With all patients taking antipsychotic medications.

### Step 4: What needs to change in order to achieve the target behavior?

To identify ways to facilitate the targeted behavior change, this step was aimed at examining the existing situation in regards to the activities of the performers. While we defined the problem as low metabolic screening and the behavioral target as psychiatrists ordering metabolic labs for people with SMI in community mental health clinics, we had to specify who would be engaging in this behavior and determine whether it was feasible for those identified to be completing the behavior. To understand this, we focused on responses from the provider focus groups and found that there were longstanding beliefs around the traditional role of a psychiatrist working in a community mental health clinic. We then utilized the TPB framework [65] to organize our focus group data into attitudes, subjective norms, and perceived behavioral control issues that might impede the target behavior (Table 1).

**Table 1:** Utilization of the theory of planned behavior (TPB) to understand barriers to having psychiatrists ordering and managing metabolic labs (target behavior)

Domains	Constructs	Barriers to target behavior
Attitudes		
Social/professional role and identity	Identity	It’s not my role to manage diabetes if I find an abnormality.
Motivation and goals	Goal setting	My patients are so sick, diabetes screening is low on the priority list
Beliefs about capabilities	Control of behavior and environment	My patients are too cognitively impaired to make it to the lab
	Self-confidence	I don’t know how to prescribe medications to treat metabolic abnormalities like diabetes
Beliefs about consequences	Outcome expectation	What if these medications to treat metabolic abnormalities cause serious adverse side effects?
Subjective norms		
Social influences	Social/group norms	Nobody else is managing diabetes!
Environmental context and resources	Resources/materials	The electronic systems are separate, so why bother?
		My medical director won’t want me to do this because we won’t be able to bill for the treatment
Perceived behavioral control		
Knowledge	Knowledge	I don’t know exactly what the ADA/APA guidelines recommend
Skills	Skills	I don’t know how to initiate medications if there are abnormalities
Environmental context and resources	Resources/materials	I don’t have reminders to get the HgA1c.
		I can’t access primary care, so why bother?

We then applied our findings to the three broad BCW components to be examined in order to achieve a target behavior: (1) capability, (2) opportunity, and (3) motivation (COM-B) (Fig. 1). The capability component refers to whether the person or persons identified as carrying out the targeted behavior change is physically and psychologically capable (e.g., knowledge, skills, stamina) of doing so. The opportunity component evaluates whether the behavior is physically accessible, affordable, socially acceptable, and able to be accomplished in a reasonable amount of time. The motivation component is defined as the intellectual processes that lead to the behavior change and includes habitual processes, emotional responses, and decision-making. These components affect one another. For example, opportunity can influence motivation as can capability; enacting a behavior can change capability, motivation, and opportunity.

We applied the TDF in this step to integrate relevant theoretical constructs to further inform the development of the current model [62]. Since each domain of the TDF correlates to a COM-B component, using the two together allows for an expansion of the COM-B components into very specific domains. Using the COM-B model and the TDF, three members of the team performed a behavioral diagnosis in order to determine what needed to change to enable psychiatrists in community mental health clinics to improve metabolic screenings among patients with SMI. The information for this behavioral diagnosis came from focus group findings, peer-reviewed literature, and research team discussion. A key factor we identified was that while psychiatrists knew how to order annual labs and had the capability to speak with their patients about the importance of getting labs, they felt uncomfortable *managing* metabolic abnormalities if these were identified through screening. We also found that lab slips were not readily available in the treatment rooms, and that psychiatrists did not have the support resources required to have these forms pre-completed for them. The behavioral diagnosis indicated that in order to achieve the target behavior of having psychiatrists order metabolic labs, there was a need for change in psychological capability, physical and social opportunity, and reflective and automatic motivation for the target behavior.

### Step 5: Identify intervention functions

Having identified the relevant COM-B components related to physician behavior that needed to change in Step 4, we explored how to address each of the barriers by focusing on specific intervention functions. Intervention functions are categories that more precisely describe routine activities. For example, “education” can include “training,” but for purposes of facilitating behavior change, it is important to distinguish between education and training with the former emphasizing the transfer of knowledge and development of understanding and the latter emphasizing the building of skills. We first prioritized the intervention functions based on the previous organization of behavior change activities (see Step 4) as any given intervention could in principle perform more than one behavior change function. Thus the intervention categories identified from the 19 existing frameworks were better conceived of as non-overlapping functions: a given intervention may involve more than one of these. Through our focus groups with psychiatrists, we learned that a many of them wanted to know when labs were due. We also found that many psychiatrist focus group participants highlighted the traditional role of psychiatrists to exclusively manage mental health, and the lack of training in managing metabolic disorders. Therefore, we explicitly identified intervention functions that would help support psychiatrists to know when labs were due and to initiate medications to treat metabolic disordered identified as a result of increased metabolic screening. Similarly, we needed to differentiate training from “modeling.” In common practice, modeling is a method used in training, but we use the term more specifically to refer to imitation of an authority figure as a motivational driver since some focus group participants voiced concern about expanding their scope of practice. A third example is the use of the term “enablement.” In everyday use, this could include most of the other intervention categories, but here the term refers to forms of enablement that are either more encompassing (as in, for example, ‘behavioral support’ for medication management) or work through other tools (as in, for example, physical and Internet-based decision support aids to provide guidance on medication dosage for specific cardiometabolic disorders).

Table 2 outlines different potential intervention functions associated with the corresponding COM-B components identified in Step 4 to facilitate the target behavior. For example, psychiatry focus group participants identified lack of reminders as a barrier to ordering labs for patients with SMI. From an intervention development perspective, this issue was understood as a barrier related to the ‘environmental context and resources’ for psychiatrists in having to navigate a new process in an already busy setting (capability and opportunity). We then selected intervention functions to help psychiatrists or other staff overcome the barriers that were most pertinent to ordering metabolic labs. Two members of the research team evaluated whether these interventions met BCW APEASE criteria (affordable, practicable, effective and cost effective, acceptable, safe, and equitable) to maximize capability, opportunity, and motivation to achieve the desired behavior change. For example, pre-completed lab slips (environmental restructuring) was selected because it met all APEASE criteria.

**Table 2:** Behavioral diagnosis and intervention functions to address change in the COM-B categories among providers and staff

COM-B component	Theoretical domains and constructs	What needs to happen for the target behavior to occur?	Potential candidate intervention functions	Potential behavioral targets (responsible staff)
Physical capability	Skills	Physical skills to prepare lab slips	Not applicable	None: psychiatrists have physical skills to prepare and distribute lab slips.
		Physical skills to distribute lab slips		
	Environmental context and resources	Lab slips need to be readily available	Environmental restructuring	Make sure lab slips are fully stocked in all treatments rooms (clinic staff).
		Psychiatrists must have access to all relevant laboratory data from the different systems in which they are served		Creation of a registry with laboratory data from several electronic records (clinic staff).
Psychological capability	Knowledge	Psychiatrists need to know and can easily learn what specific metabolic labs to order	Education	Education about metabolic screening guidelines (primary care consultant).
				Education about medications (and side effects) to treat potential metabolic abnormalities (primary care consultant).
			Persuasion	Using colorful and readable visual charts to motivate learning the cutoffs for different normal cardiometabolic levels (primary care consultant creates; clinic staff distributes).
		Psychiatrists need to know how to initiate treatment when metabolic abnormalities are identified	Training	Receive instruction on how to read and use the decision charts with algorithms in making treatment decisions (primary care consultant).
	Memory	Psychiatrists need to remember the algorithms for treatment	Enablement	Making algorithm decision charts readily available by distributing copies to all psychiatrists, posting copies in all treatment rooms, and making it accessible electronically (primary care consultant creates; clinic staff distributes).
	Attention and decision processes	Psychiatrists need to have support for treatment decisions	Environmental restructuring	Providing access to a primary care consultant for clinical decision support through the electronic medical record (EMR) system (IT administrator).
	Social role and identity	Psychiatrists need to believe that it is their role to screen and treat metabolic abnormalities.	Modeling	Medical director participates in trainings and uses algorithms and primary care consultant via EMR system for decision support around managing cardiometabolic lab results (clinic medical director).
Physical opportunity	Intentions and goals	Patients need to receive filled out lab slips from psychiatrists.	Enablement	Provide psychiatrists with completed lab slips monthly for patients with labs due and samples of completed lab slips in examination rooms; ensure that examination rooms are fully stocked with lab slips (clinic staff).
		Utilize phlebotomy services that are located near clinic.	Persuasion	Distribute map of identified lab screening locations and transportation route to all patients with labs due to increase motivation to follow through on obtaining labs (clinic staff).
		Patients who are disorganized or have physical disabilities should receive assistance to obtain phlebotomy services	Environmental restructuring	Ensure the availability of a peer navigator as a physical resource for assistance with patients that require assistance in obtaining labs (peer navigator).
Social opportunity	Social influences	Staff psychiatrists observe senior health providers ordering and managing metabolic labs.	Modeling	Local clinic medical director participates in and helps with designing the intervention (clinic medical director).
		Psychiatrists need support to manage abnormalities and access to primary care services	Enablement	The intervention has the support of local champions and leadershipin the form of additional resources that aid psychiatrists in managing cardiometabolic labs (clinic medical director).
Reflective motivation	Optimism	Psychiatrists need to believe that regular metabolic lab screening and treatment will lead to better care	Education	Provide education about improved health outcomes after screening and treatment, and give examples from prior studies to show that it is possible for patients with SMI to have metabolic labs managed in community mental health settings (primary care provider).
	Beliefs about consequences	Psychiatrists need to believe that their work will decrease mortality rates among this population	Persuasion	
Automatic motivation	Reinforcement	Need an established routine for reminding psychiatrists about labs and providing feedback for following through on labs.	Enablement	Automated system for reminding psychiatrists which patients have labs due (IT administrator).
			Incentivization	Provide regular performance monitoring to show proportion of patients for each provider that receive lab draws over time and reward providers in their efforts to order lab draws in their patients (IT administrator)
			Education	Provide information regarding improved health outcomes for patient population (primary care consultant).

### Step 6: Identify policy categories

After developing the intervention strategy, we evaluated what policies will support the delivery of the intervention functions in this step. Seven policy categories to help support and enact the interventions were considered, including communication/marketing, guidelines, fiscal measure, regulations, legislation, environmental/social planning, and service provision. We identified which of the seven policy categories were most applicable to the identified intervention function (Table 3). For example, we decided to brand the intervention by creating a logo and placing it on printed decision charts to create a culture change and used branded mugs and birthday cards to staff members as persuasion.

**Table 3:** Policy categories for the CRANIUM collaborative care model

Intervention function	Policy category	Candidate policies to support the delivery of the intervention functions
Education	Guidelines	Treatment protocols for management of metabolic disorders were distributed (on-line and laminated).
Persuasion	Communication/marketing	Mugs and birthday cards with logo for clinic staff; logo on algorithms
Incentivization	Fiscal measures	Treats (e.g., cookies) were provided to the team with the highest metabolic screening rates.
Coercion	Service provision	Treatment teams knew which teams were the “best” and might be coerced to compete
Training	Guidelines	A primary care physician reviewed guidelines and protocols for management of metabolic disorders.
	Service provision	Established a support service of a primary care consultant for psychiatrists to access on-line
Environmental restructuring	Environmental/social planning	Restructuring the clinic to include in pre-completed lab slips in all interview rooms.
		Stepped care approach where peer navigators could assist patients in going to phlebotomy services.
Modeling	Service provision	Medical Director adopts behavior change and becomes the champion and role model for other staff.
Enablement	Environmental/social planning	Changing roles where psychiatrist can safely initiate treatment of common metabolic abnormalities.

### Step 7: Identify behavioral change techniques

After selecting the intervention functions and policy categories that might help deliver the intervention, we identified behavioral change techniques to develop the final model. A behavioral change technique is defined as “an active component of an intervention designed to change behavior” [62]. The techniques are active ingredients within the intervention and leads to observable and replicable behavior change. Two members of the research team jointly determined the following behavioral change techniques most relevant for this model based on the results of the focus groups from the stakeholder interviews and then independently reviewed by research team members and ultimately agreed upon in a group consensus. Furthermore, the following identified behavior change techniques are thought best to serve the previously identified intervention functions that were linked to the BCW components:

- Additional resources: psychiatrists would need additional resources to ease the process of ordering metabolic labs, specifically a monthly registry of patients who are due for screening labs and pre-completed laboratory slips.
- Social support: two new team members will provide social support: (1) peer navigator to help complete lab slips, assist patients to phlebotomy services, and enter data into the electronic medical record, and (2) a primary care consultant to help provide clinical decision support for psychiatrists initiating medications to treat metabolic abnormalities.
- Goal setting: regular performance monitoring will help ensure that 80% of all patients receive annual metabolic screening.
- Problem solving: psychiatrists will have immediate electronic access to a primary care consultant to provide clinical decision support.
- Action planning: algorithms help provide psychiatrists with a plan for any abnormal values identified on screening metabolic labs.
- Self-monitoring: performance monitoring of metabolic screening status on the panel of each individual psychiatrists.
- Review of behavior and outcome goals: conduct a quarterly panel review for all patients with labs due over a three-month period to troubleshoot complex cases and to receive feedback from psychiatrists and ancillary staff on the intervention.

To achieve the targeted behavior change of having psychiatrists order labs and to manage metabolic abnormalities, it was critical to provide them with support and the information they needed to do the task, and the expected health consequences of *not* making this the behavior change. In addition, having regular and timely feedback sessions on the outcomes of the behavior lead to self-monitoring, problem-solving, and further action planning.

### Step 8: Identify mode of delivery

**Fig. 2:**
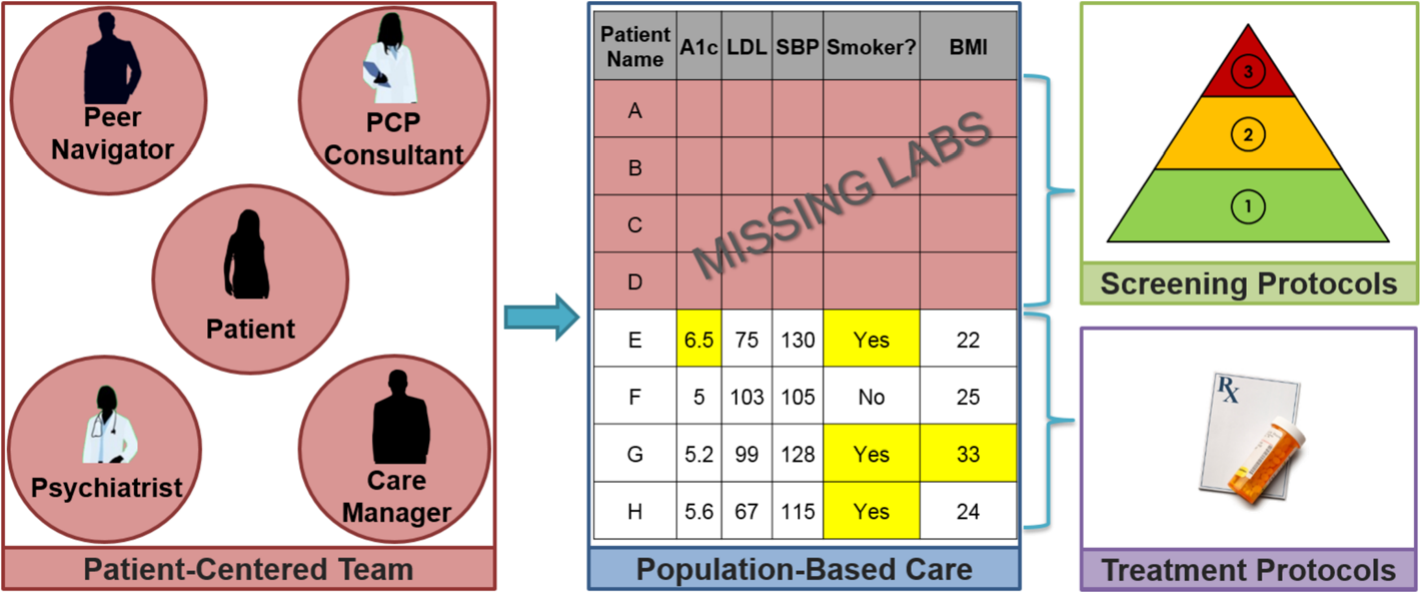
CRANIUM intervention components

In specifying the behavioral change techniques and focusing on psychiatrist behavior, we then identified potential modes of delivering the intervention and created an integration of care model that we called CRANIUM (cardiometabolic risk assessment and treatment through a novel Integration model for underserved populations with mental illness). CRANIUM is comprised of four components: patient-centered team care (psychiatrist, case manager, primary care provider, and peer navigator), population-based care (patient registry), screening protocols (HgA1c, LDL, SBP, DBP), and treatment protocols (guideline-recommended pharmacological management for diabetes, hypertension, and dyslipidemia) (Fig. 2). In addition to the member of a usual community mental health care team (psychiatrist and care manager), the new patient-centered team includes a primary care consultant and a peer navigator. Because patients with mental illness receive their primary care from various health care settings—and laboratory data are not integrated with the behavioral health EMR, we had to identify a means to provide this information to their patient-centered team. As such, electronic registry that consolidates information from multiple electronic medical records (EMRs) was developed which had pertinent laboratory and vital sign data. Psychiatrists received this monthly registry containing annual metabolic screening results on all of their patients. Although registries like this are common in primary care, having easily accessible, electronic, and timely laboratory data on health monitoring is a rarity in public mental health care systems [84]. A quarterly panel management meeting was proposed to focus on of patients who are missing screening labs and to develop individualized support plans based on screening protocols. Among those with identified cardiometabolic risk factor abnormalities, treatment protocols with decision support tools were developed. These protocols include reminders to encourage smoking cessation, obesity care, and offer evidence-based pharmacologic treatment for identified CVD risk factor abnormalities.

## Discussion

**Fig. 3:**
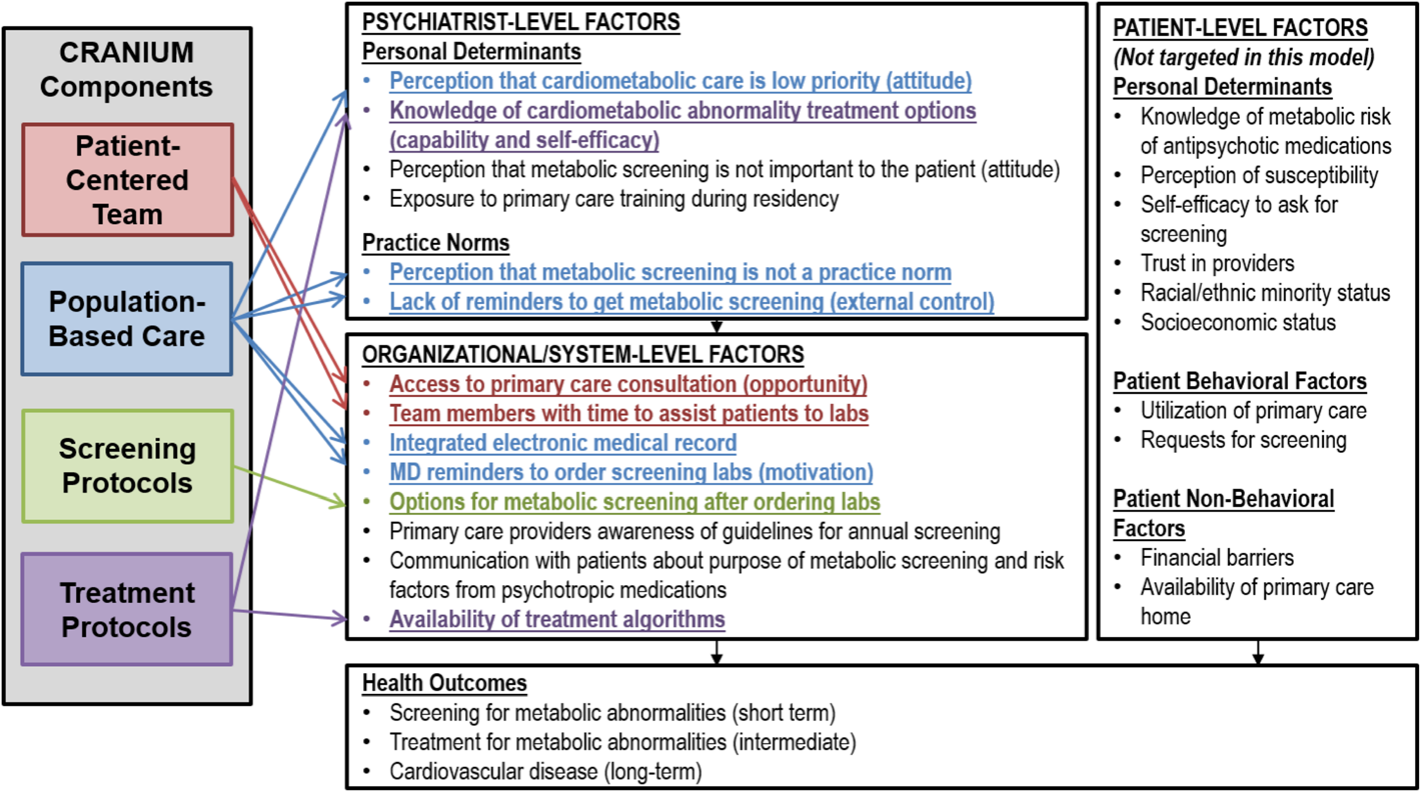
CRANIUM targets to improve cardiometabolic screening and treatment in community mental health clinics

By using the results from application of the BCW framework, we were able to systematically design an integration of care model to increase screening and initial treatment of CVD risk factors in people with SMI that was grounded in behavior change theory. We used both provider-level and system-level targets to build this model (Fig. 3).

The model that emerged was an integrated and patient-centered care model to improve healthcare delivery for individuals with SMI and incorporates principles from the several prior models [85–88]. The model was called CRANIUM. The CRANIUM intervention includes elements of patient-centered team care, population-based care, screening protocols, and evidence-based treatment protocols. This model utilized several BCW intervention functions (e.g., environmental restructuring, education, training, enabling, and modeling) to address the cultural shift required to change the behavior of community psychiatrists. The template for intervention description and replication (TIDieR) checklist [89] was used to describe specific components of the intervention (Appendix C).

CRANIUM bears the most similarity to the evidence-based Collaborative Care model that is based in primary care settings [90]. Since 40% of people with SMI regularly access community mental health services [91] and these settings are de-facto health home for these patients, CRANIUM attempted to resolve a systemic problem by leveraging technology to enable community psychiatrists to take a more active role in screening and initial management of metabolic disorders. Utilizing psychiatrists to take on this role represents a significant culture shift, and also requires a complex system change to alleviate structural dysfunctions between mental health and primary care. Utilizing the BCW framework, we were able to develop a model that has a strong foundation in behavioral theory and hopefully will be highly acceptable to end-users.

The process of developing CRANIUM included many strengths. First, the use of the BCW and TDF frameworks are heavily grounded in behavior change theory and linked to evidence-based intervention functions that can orient an intervention to a targeted setting and population. As mentioned previously, only one other study focused on integration of care in a recent systematic review of changing healthcare professionals’ behavior [68], with ours being the only to formally identify and link barriers to integration of care to specific intervention components. Second, by using the BCW framework to defined the behavioral target to address the specific problem of low adherence to national guidelines for metabolic screening [4], our model is the first to focus on guideline adherence that requires integration of care and co-management by physicians [76]. As such, this paper adds significant value to use of such behavioral frameworks when planning integration of care activities across medical disciplines.

Finally, the use of feedback from multidisciplinary staff in quarterly meeting during the course of the intervention design was a unique way to maximize community engagement.

However, there are limitations to using these approaches [92], for example, they require a lengthy barrier identification and intervention planning process, and it is often not possible to address all of the barriers and potential enablers identified, making it difficult to actualize all of the components that can aid in addressing the behaviors required to make multi-level changes. Alternatively, we could have applied other implementation science frameworks such as CFIR or RE-AIM [93, 94]. These might have been helpful to place/map the intervention into the context of clinic or community settings, but would not have provided the same level of focus on behavior change which we believed was critical for this problem. Another limitation is that the overall approach was primarily aimed at targeting the individual health care provider, but did not necessarily focus on case management teams or the larger public health system.

## Conclusions

Individuals with SMI experience a highly fragmented system of care, contributing to poor health outcomes. The utilization of the Behavior Change Wheel Framework enabled a systematic and theory-driven approach to be taken to the development of a behavior change intervention within clinical practice for the management of metabolic disorders by community psychiatrists. To our knowledge, no one has used this framework to develop an intervention to improve the health of people with severe mental illness in community mental health clinics. Public health administrators might consider using these systematic frameworks in addressing some of their most complex systems-level problems. In our opinion, this framework proved to be a practical way of using theory to inform the development of an evidence-based integration of care solution that may result in significant public health implications.

## Appendix A

### Guiding questions for focus groups

For all focus groups

- Describe any barriers that might prevent people with mental illness from receiving annual screening for metabolic disorders.
- Describe any barriers people with severe mental illness might have in getting “medical” medications (e.g., prescribing a statin for high cholesterol).
- Are there any barriers that might be specific to racial or ethnic minority populations?
- What would you think about psychiatrists providing these medical medications *if* they had the support of a “virtual” (email) primary care physician (PCP) consultant?
- What are your concerns about having a virtual primary care consultant being used to facilitate treatment of high cholesterol by psychiatrists?
- What are your thoughts about having pre-made algorithms for common disorders (e.g., high cholesterol) for psychiatrists to use?

For provider focus groups only

- Please describe your current practices for metabolic screening and treatment of metabolic abnormalities.

For patient focus groups only

- Have you been treated for metabolic complications by your psychiatrist? If so, which one (blood pressure, diabetes, dyslipidemia)?

## Appendix B

**Table 4:** Focus group participant demographic information

	All participants (*n* = 29)	Psychiatrists (*n* = 8)	Primary care (*n* = 6)	Administrators (*n* = 7)	Consumers (*n* = 8)
Age in years, mean (range)	46.6 (28–67)	44.5 (35–67)	44.8 (32–52)	49.1 (41–58)	48 (range 28–65)
Sex					
Male	14 (48.3%)	3 (37.5%)	2 (33.3%)	3 (43%)	6 (75.0%)
Female	5 (51.7%)	5 (62.5%)	4 (66.7%)	4 (57.1%)	2 (25.0%)
Race					
African American	3 (10.3%)	1 (12.5%)	0 (0%)	0 (0%)	2 (25.0%)
Latino	5 (17.2%)	0 (0%)	0 (0%)	2 (28.6%)	3 (37.5%)
White	13 (44.8%)	6 (75.0%)	2 (33.3%)	2 (28.6%)	3 (37.5%)
Asian	7 (24.1%)	1 (12.5%)	3 (50.0%)	3 (42.9%)	0 (0%)
Native American	0 (0%)	0 (0%)	0 (0%)	0 (0%)	0 (0%)
Other/multiracial	1 (3.4%)	0 (0%)	1 (16.7%)	0 (0%)	0 (0%)
Years worked in health or mental health, mean (range)	n/a	12.9 (4–30)	15.3 (3–25)	23.6 (17–33)	n/a

## Appendix C

**Table 5:** The TIDieR (template for intervention description and replication) checklist

Item #	Item	Where located	
		Primary paper page	Other
1	Brief name: CRANIUM (cardiometabolic risk assessment and treatment through a novel integration model for underserved populations with mental illness). The CRANIUM intervention includes the following elements: patient-centered team care, population based care, screening protocols and evidence-based treatment protocols.	Page 15	http://cranium.ucsf.edu/
2	Why: changing the behavior of community psychiatrists to initiate treatment of cardiometabolic risk factors was essential for the success of this intervention. As such, we relied heavily on several theoretical framework to develop the intervention (The Behavior Change Wheel, Theoretical Domains Framework, and the theory of planned behavior). We also addressed organizational-level factors that could facilitate the behavior change.	Pages 5–7, Fig. 3	
3	What (material): 3a. Patient-centered team-care: each team was provided with roles/responsibilities of each team member. 3b. Population-based care: a patient registry and pre-completed lab slips were provided to teams. 3c. Screening protocols: we based our screening protocols on the 2004 APA/ADA guidelines. 3d. Evidence-based treatment protocols: protocols were placed in all treatment rooms and provided electroinically to psychiatrists.	Pages 13–14 and Fig. 2	3a. http://tinyurl.com/ydarwyry 3b. http://tinyurl.com/y9cjatjd 3c. https://www.ncbi.nlm.nih.gov/pubmed/14747245 3d. http://tinyurl.com/y9fxwg8v
4	What (procedures): 4a. Patient-centered team-care: all team members meet quarterly to discuss patients. The ePCP is also available for as needed questions online 4b. Population-based care: psychiatrists and case managers received monthly registries on metabolic screening of their patient panels. 4c. Screening protocols: psychiatrists receive a lecture on screening protocols. All team members reviewed patients missing labs at quarterly panel management meetings 4d. Evidence-based treatment protocols: all psychiatrists received a 1× lecture on treatment and discussed patients needing treatment at the quarterly panel management meetings.	Page 14–15	4c and 4d. http://cranium.ucsf.edu/article/tools-clinicans
5	Who provided: the psychiatrists receieved a 1× training on treatment of cardiometabolic risk factors by the primary care consultant. The case manager and peer navigator were taught about panel management.	Page 13–15	http://tinyurl.com/ydarwyry https://youtu.be/v1G4BuklmbA
6	How: the training of psychiatrists on treatment of carediometabolic risk factors was online and/or in person. The panel management meetings were in-person, group meetings. The support from the primary care consultant was individual and delievered electronically, on the phone, or in-person.	Page 13–15	http://cranium.ucsf.edu/article/tools-clinicans
7	Where: the intervention itself occurred in the community mental health clinic. The panel management meetings happened quarterly in a clinic conference room	Page 10	
8	When and how much: 4a. Patient-centered team-care: all team members met quarterly to identify patients missing labs and/or needing treatment (4c). 4b. Population-based care: psychiatrists and case managers received monthly registries on metabolic screening of their patient panels. All team members reviewed patients missing labs and patients needing treatment at quarterly panel management meetings 4c. Screening protocols: psychiatrists receive a one-time lecture on screening protocols. 4d. Evidence-based treatment protocols: all psychiatrists received a one-time lecture on treatment and discussed patients needing treatment at the quarterly panel management meetings. The ePCP was also available for as needed via phone or email to discuss psychiatrists’ questions about treatment	Page 13–14	
9	Tailoring. the intervention was tailored by the opinions of the patients, providers, and administrators. We do not currently know what additional tailoring might be avialble at other sites.	Page 8	
10	Modificiations	N/A^¥^	N/A^¥^
11	How well (planned)	N/A^¥^	N/A^¥^
12	How well (actual)	N/A^¥^	N/A^¥^

^¥^Not applicable. As per TIDieR checklist, these items are not relevant and cannot be described until the study is complete
